# Effects of sesamin on chondroitin sulfate proteoglycan synthesis induced by interleukin-1beta in human chondrocytes

**DOI:** 10.1186/s12906-017-1805-1

**Published:** 2017-05-31

**Authors:** Warunee Srisuthtayanont, Dumnoensun Pruksakorn, Prachya Kongtawelert, Peraphan Pothacharoen

**Affiliations:** 1 Department of Biochemistry, Thailand Excellence Center for Tissue Engineering and Stem Cells, Faculty of Medicine, Chiang Mai University, 110 Intavaroros Road, Sripoom, Muang, Chiang Mai, 50200 Thailand; 2 Department of Orthopedics, Orthopedic Laboratory and Research Netting Center (OLARN Center), Faculty of Medicine, Chiang Mai University, Intavaroros Road, Sripoom, Muang, Chiang Mai, 50200 Thailand; 3 Excellence Center in Osteology Research and Training Center (ORTC), Chiang Mai University, Intavaroros Road, Sripoom, Muang, Chiang Mai, 50200 Thailand

**Keywords:** Sesamin, Chondroitin sulfate proteoglycan, Human chondrocyte, Interleukin-1beta

## Abstract

**Background:**

Numerous studies have reported on the health benefits of sesamin, a major lignin found in sesame (*S. indicum*) seeds. Recently, sesamin was shown to have the ability to promote chondroitin sulfate proteoglycan synthesis in normal human chondrocytes. This study assesses the anti-inflammatory effect of sesamin on proteoglycans production in 3D chondrocyte cultures.

**Methods:**

To evaluate the effects of sesamin on IL-1β-treated human articular chondrocytes (HAC) pellets, the pellets were pre-treated with IL-1β then cultured in the presence of various concentrations of sesamin for 21 days. During that period, the expression of IL-1β, glycosaminoglycans (GAGs) content and Chondroitin sulfate proteoglycans (CSPGs) synthesis genes (*ACAN, XT-1, XT-2, CHSY1* and *ChPF*) was measured. The GAGs accumulation in the extracellular matrix was determined on day 21 by histological analysis.

**Results:**

There was clear evidence that sesamin upregulated expression of all the CSPGs synthesis genes, in contrast to the down-regulation of IL-1β expression both in genes and in protein levels. The level of release and matrix accumulation of GAGs in IL-1β pre-treated HAC pellets in the presence of sesamin was recovered. These results correlate with the histological examination which showed that sesamin enhanced matrix CSPGs accumulation.

**Conclusions:**

Sesamin enhances CSPGs synthesis, suppresses IL-1β expression and ameliorates IL-1β induced inflammation in human chondrocytes. Sesamin could have therapeutic benefits for treating inflammation in osteoarthritis.

## Background

Osteoarthritis (OA) is the most common degenerative joint disease and is associated with a risk of reduced mobility. The disease occurs worldwide, usually in the elderly, where it results in an especially high economic burden. This disease causes movement to be painful, reducing quality of life and increasing the risk of other diseases such as diabetes and high blood pressure. OA not only affects health, but also creates social and economic problems because patients lose the ability to conduct routine activities and also incur high medical charges [1].

Loss of homeostasis due to an imbalance between anabolic and catabolic processes is driven by cytokine cascades combination with the inflammatory mediators, the key event occurring in cartilage during pathogenesis. An increase of inflammatory cytokines, mainly interleukin-1β (IL-1β), is found in chondrocytes as well as synoviocytes of OA patients. These in turn decrease proteoglycans production and collagen synthesis while increasing catabolic factors including matrix metalloproteinase (MMP) and other inflammatory mediators such as IL-8, IL-6, prostaglandin E_2_ and nitric oxide (NO) synthesis which promote OA [2]. Current OA therapies aim to manage chronic pain and improve joint function. NSAIDS are used mainly as medication for OA treatment, but they have many adverse effects and the therapeutic mechanism of NSAIDS is not directed to the underlying disease pathogenesis. Systemic slow acting drugs (SYSAD) such as glucosamine sulfate/chondroitin sulfate, hyaluronan and diacerine are also used because of their contribution to delaying disease progression by catabolic process inhibition and favor the anabolic process [3].

Many phytochemicals with anti-inflammation effects are claimed to be chondroprotective agents and are of interest as an alternative choice for OA treatment. Sesamin, a major lignin found in sesame seeds, has been reported to have health benefits. Reported pharmacological properties of sasamin include anti-inflammation [4], anti-oxidant [5], anti-hypertensive, inducing apoptosis in cancer cells [6], lowering blood cholesterol, improving fatty acid metabolism [7] and neuroprotective effect against hypoxia [8]. A recent study described the chondroprotective effect of sesamin on normal human articular chondrocytes (HAC) works by promoting matrix sulfated glycosaminoglycans (GAGs) content and upregulation of the chondroitin sulfate proteoglycan (CSPGs) synthesis genes: *ACAN, XT-1, XT-2, CHSY1* and *ChPF* resulting in a protective affect against OA [9]. In this study we evaluated the effect of sesamin on pathological HAC in pellet culture in which the inflammation process in OA pathogenesis had been induced by IL-1β.

## Methods

### Materials

Primary human articular chondrocyte (HAC) was isolated from non-osteoarthritic joints taken from normal cartilage of 2 male and 2 female patients aged between 18 and 45 years at Maharaj Nakorn Chiang Mai Hospital. The isolated chondrocytes were cultured in Dulbecco’s modified Eagle’s medium (DMEM) containing 10% fetal bovine serum (FBS) as standard protocol.

The seeds of *Sesamum indicum* Linn. were collected from Lampang province of Thailand and voucher specimen (BKF no. 138181) has been deposited at the National Park, Wildlife and Plant Conservation Department, Ministry of Natural Resources and Environments, Bangkok, Thailand. The sesamin was purified from sesame seeds using the method described by T. Phitak *et al.* [4].

For western blot analysis, a goat anti-human aggrecan G1-IGD-G2 Domains anti-body (Cat no. AF1220) was purchased from R&D system. Mouse anti-MMP13 monoclonal antibody (Cat no. MAB3321) and rabbit anti-ADAMTS-4 antibody (Cat no. ABT178) were purchased from Merck Millipore.

### Chondrocyte expansion and 3D culture

Primary chondrocytes were expanded by monolayer culture and grown to confluence in DMEM containing 10% FBS in a humidified incubator with 5% CO_2_ at 37 °C. The chondrocytes (passage 3) in the monolayer culture were used to create pellets. The 5 × 10^5^ trypsinized cells were centrifuged at 160 g for 5 min and cultured with 500 μl chondrogenic medium (10% FCS/DMEM, Insulin-Transferrin-Selenium (ITS 1×), 25 μg/ml ascorbic acid 2-phosphates and 10^−7^ M dexamethasone) in 15 ml conical tubes. Pellets were grown in a humidified incubator with 5% CO_2_ at 37 °C.

### Optimization of IL-1β concentration for treated pellets

Pellets were treated with IL-1β 0-10 ng/ml on the day of pellets formed into spherical shape then incubated for 3 days. After that, the pellets were cultured with IL-1β free media for 21 days. Culture mediums and pellets were collected at intervals for measurement of GAGs release and gene expression.

### Western blot analysis

Pellet lysates (80 mg protein/lane) were subjected to 12% gel SDS-PAGE and transferred to nitrocellulose membrane. Membranes were blocked with 5% milk in PBS/Tween20, then incubated with specific antibodies. The interested protein bands were developed using Supersignal West Femto Substrate (Thermo Scientific) and photographed using the molecular chemidoc XRS system and Gel-Doc machine (Bio-Rad). Band density was calculated using the Totallab TL120 software.

### Investigation the effect of sesamin on IL-1β treated pellets

Groups of pellets were either pre-treated with 10 ng/ml IL-1β for 3 days or left untreated. Both groups were then cultured with sesamin 0-1 μM for further 21 days. Culture mediums were collected to measure GAGs release and pellets were collected to measure gene expression, GAGs content and DNA concentration.

### Sulfated glycosaminoglycans content in culture media and pellet matrix

The GAGs release from the HAC pellets and GAGs accumulation were measured in culture media and papain digested pellets by DMMB assay [9]. Matrix GAGs accumulation in pellet sections was investigated using safranin O staining.

### Analysis of IL-1β concentration in culture media

Culture media or standard IL-1β (0-500 pg/ml) 100 μl were added to mAb against IL-1β in wells pre-coated with human IL-1β, mixed and covered by a plate cover sheet. All were incubated on an orbital shaker (200 rpm) at room temperature. After incubation for 3 h., the plate cover was removed and the culture media was discarded. The wells were washed 6 times; after the final wash, the plate was dried on tissue paper. TMB substrate solution was added to the wells (100 μl/well) and the plate was then incubated in the dark for 10 min. The reaction was stopped by adding 100 μl/well of stop solution. Light absorbance at 450 nm. was measured with a micro plate reader spectrophotometer. The concentration of IL-1β was calculated using the genesis program with reference to the standard curve.

### Gene expression quantitation

HAC culture pellets were collected and RNA was extracted using an RNA extraction kit (GE Healthcare) following the manufacturer’s protocol. A total RNA (0.25 μg) was converted to cDNA using a BIOLINE SensiFAST™ cDNA synthesis kit following the mixer and reaction protocol. Real-time PCR was performed to investigate gene expression using a BIOLINE SensiFAST™ SYBR® No-ROX Kit. The primers were purchased from Invitrogen (Table 1). The level of gene expression of the samples was compared with that of *GADPH* (the house-keeping gene) calculated using the 2^-ΔΔCT^ method [10].

**Table 1:** Real-Time PCR Primers sequences

Gene	Real-time PCR primer sequence (5′-3′)
*ACAN*	Forward: ACTTCCGCTGGTCAGATGGA Reverse: TCTCGTGCCAGATCATCACC
*XT-1*	Forward: GTGGATCCCGTCAATGTCATC Reverse: GTGTGTGAATTCGGCAGTGG
*XT-2*	Forward: CGAATCGCCTACATCATGCTGG Reverse: TAAACGGCCTTGAGGAGACG
*CHSY-I*	Forward: CCCGCCCCAGAAGAAGTC Reverse: TCTCATAAACCATTCATACTTGTCCAA
*ChPF*	Forward: AGATCCAGGAGTTACAGTGGGAGAT Reverse: CCGGGCGGGATGGT
*IL-1β*	Forward: AAACAGATGAAGTGCTCCTTCCAGG Reverse: TGGAGAACACCACTTGTTGCTCCA
*MMP-13*	Forward: TTGAGCTGGACTCATTGTCG Reverse: GAGCCTCTCAGTCATGGAG
*ADAMTs-4*	Forward: CCCCAGACCCCGAAGAGCCA Reverse: CCCGCTGCCAGGCACAGAAG
*DEC*	Forward: CCTGATGACCGCGACTTCGAG Reverse: TTTGGCACTTTGTCCAGACCC
*GADPH*	Forward: GAAGGTGAAGGTCGGAGTC Reverse: GAAGATGGTGATGGGATTTC

### Statistical analysis

Estimated values are given as mean ± standard deviation from triplicate samples of each of three independent experiments. One-way ANOVA and LSD post hoc test were used to compare control with IL-1β treated pellets and to compare IL-1β pre-treated pellets alone with IL-1β pre-treated pellets with sesamin. Values were considered significant at *p* < 0.05.

## Results

### The effect of IL-1β on anabolic and catabolic gene expression

**Fig. 1:**
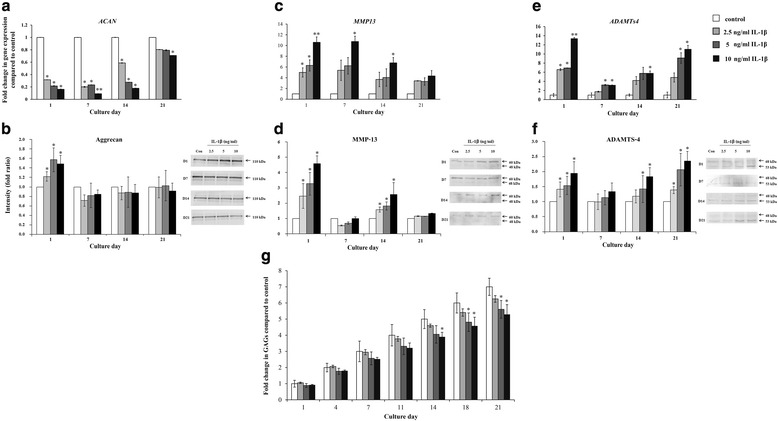
Effects of various IL-1β concentrations (2.5 to 10 ng/ml) pre-treatment on the gene and protein expression level of aggrecan (**a**, **b**), MMP-13 (**c**, **d**) and ADAMTS-4 (**e**, **f**) of HAC pellets. GAGs released in media during 21 days of culture with various concentrations of IL-1β (0 to 10 ng/ml) in 3-day pre-treated HAC pellets (**g**). The data presented are the mean ± S.D. of 3 independent cultures. Statistical significance compared to the untreated (control) * = *p* < 0.05, ** = *p* < 0.01

To investigate chondrocyte metabolic alterations of IL-1β on anabolic and catabolic gene expression, HAC pellets were pre-treated with various concentrations of IL-1β (0-10 ng/ml) for 3 days and cultured in IL-1β-free medium for a further 21 days. The cell lysate was harvested and purified for total RNA. The level of anabolic (*ACAN*) and catabolic (*MMP-13, ADAMTs4*) mRNA were measured by Real-Time RT-PCR at days 1, 7, 14 and 21 after 3 days of IL-1β pre-treatment. The protein expression of aggrecan, MMP-13 and ADAMTS-4 were measured by western blot analysis. All concentrations of IL-1β treatment (2.5-10 ng/ml) showed the ability to decrease expression of *ACAN* (Fig. 1a) and increased aggrecan on day 1. The increase of gene expression of *MMP-13* (Fig. 1c) and *ADAMT*s4 (Fig. 1e) were observed compared to the control. A 3-day pre-treatment with 10 ng/ml IL-1β was able to significantly suppress *ACAN* gene expression while increased *MMP-13* and *ADAMTs4* gene expression compared to the control. On day 1 after IL-1β treatment, the protein level of aggrecan was increased by concentration dependent manner when compared to the control. On day 7 throughout day 21, IL-1β did not affect the aggrecan level when compared to the control (Fig. 1b).

A single 10 ng/ml dose of IL-1β significantly downregulated *ACAN* and upregulated *ADAMTs4* expression throughout the 21 day culture period. Significant induction of *MMP-13* expression was observed only for 14 day after IL-1β treatment; on day 21, induction was slightly increased compared to the control.

A 3 day pre-treatment with 10 ng/ml IL-1b significantly increased MMP-13 protein expression on day 1 and day 14 when compared to the control. The induction of ADAMTS-4 protein was observed on day 1,14 and 21 when pre-treated pellet with 10 ng/ml IL-β.

For that reason, this concentration was selected for use in treating pellets during further investigations.

### The effect of IL-1β on GAGs release in culture media

The effect of IL-1β on GAGs production was determined by pre-treatment of HAC pellets with various concentrations of IL-1β (0-10 ng/ml) for 3 days followed by further culture in IL-1β-free medium for 21 days. Culture media was collected every 2 to 3 days for GAGs release measurement by DMMB assay. Levels of GAGs in culture media decreased in HAC pellets pre-treated with IL-1β in a dose and time dependent manner when compared to the control (Fig. 1d). Significant decreases of GAGs levels were observed at days 14 and 21. At the highest concentration of IL-1β (10 ng/ml), GAGs released from the HAC pellets significantly decreased beginning at day 14. This finding indicates that the HAC pellets pre-treated with 10 ng/ml of IL-1β had the ability to suppress GAGs production throughout the 21 day period of pellet culture. Decreases in GAGs release in response to IL-1β pre-treatment were related to decreases of *ACAN* gene expression (Fig. 1a). Overall, the data indicate that a concentration of IL-1β of 10 ng/ml is the optimal dose to stimulate alteration of the anabolic process of CSPGs in HAC pellets.

### Effect of sesamin on *IL-1β* expression in IL-1β induced HAC pellets

**Fig. 2:**
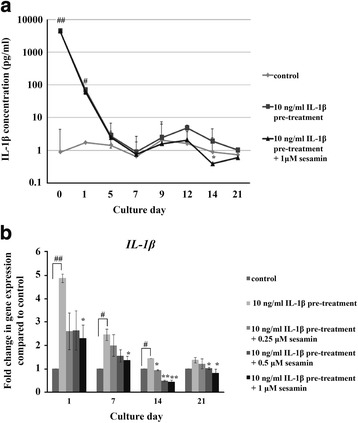
Effects of IL-1β and sesamin on the level of IL-1β in the culture media of 3 day IL-1β pre-treated HAC pellets (**a**). The effects of sesamin on IL-1β gene expression in IL-1β pre-treated HAC pellets (**b**). Data are the mean ± S.D. of three independent cultures. Statistical significance of IL-1β pre-treatment alone vs untreated (control): # = *p* < 0.05, ## = *p* < 0.01 and sesamin treatment vs IL-1β pre-treatment alone: * = *p* < 0.05, ** = *p* < 0.01

To demonstrate that a single pre-treatment of 10 ng/ml IL-1β is sufficient to initiate the alteration of HAC metabolism for the 21 days of the HAC pellet culture period, the following procedures were followed. The IL-1β level in the medium of the HAC pellet culture was mesured after a washout period of 3 days following pre-treatment with 10 ng/ml IL-β. After washout, the level of IL-1β was detectable in the culture medium (approximately 0.8 ng/ml) after day 1, rapidly decreasing through day 7. The level of IL-1β in culture medium slightly increased from day 9 to day 12 then decreased from day 14 to day 21. A low concentration of IL-1β (range: 0.5 to 2 pg/ml) was present in the culture medium of untreated HAC pellets for the entire 21 days of culture. The rise in IL-1β concentration in the medium was significantly higher in the IL-1β pre-treatment group (Fig. 2a).

**Fig. 3:**
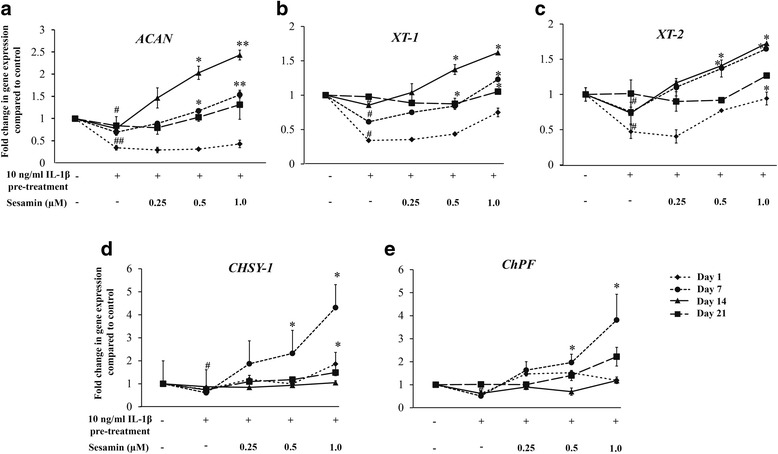
Effects of sesamin on CSPG synthesis gene expression in IL-1β pre-treated HAC pellets over 21 days: *ACAN* (Panel **a**), *XT-1* (Panel **b**), *XT-2* (Panel **c**), *CHSY1* (Panel **d**) and *ChPF* (Panel **e**). Data presented are the mean ± S.D. of three independent cultures. Statistical significance in comparison of IL-1β pre-treatment alone vs untreated (control): # = *p* < 0.05 and in comparison of sesamin treatment vs IL-1β pre-treatment alone: * = *p* < 0.05

The effect of sesamin on IL-1β release was further investigated by pre-treating pellets with 10 ng/ml IL-1β HAC for 3 days followed by treatment with 1 μM of sesamin for 21 days. During the 21 day culture period, the level of IL-1β in the culture medium was measured. The effects of sesamin on IL-1β release from IL-1β pre-treated HAC pellets were clearly seen at days 14 and 21. Sesamin decreased IL-1β release levels from day 14 through day 21 (Fig. 3a) compared to pellets pre-treated with IL-1β alone. To confirm the protein expression level results, the effect of sesamin on IL-1β gene expression was also determined. The 3 day pre-treatment with IL-1β induced significant IL-1β gene expression from day 1 through day 14 compared to the control. The level of IL-1β gene expression decreased in a time dependent manner. In the presence of sesamin, IL-1β gene expression was reduced compared to IL-1β pre-treatment alone. Sesamin at 1 uM significantly supressed IL-1β gene expression which was related to the IL-1β release level (Fig. 2b).

### Effect of sesamin on CSPGs synthesis gene expression in IL-1β-induced HAC pellets measured using real-time RT-qPCR

**Fig. 4:**
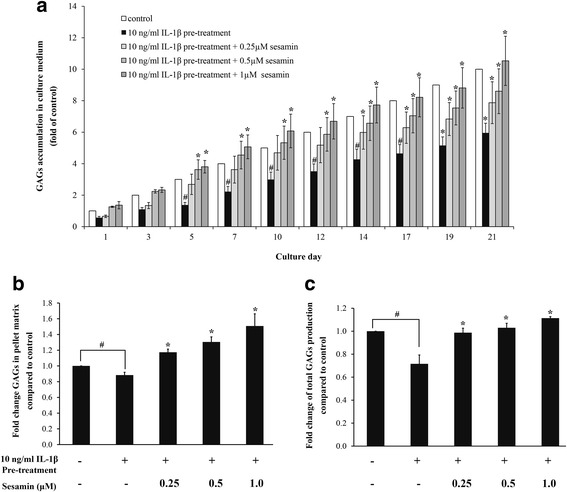
The effects of sesamin on GAGs production in IL-1β 3-day pre-treated HAC pellets. **a** Effects of sesamin on GAGs secretion to the culture medium over 21 days; **b** GAGs accumulation in the pellet matrix after 21 days treatment; and **c** total GAGs production after 21 days treatment. Data presented are the mean ± S.D. of three independent cultures. Statistical significance of the difference between IL-1β pre-treatment alone and untreated (control): # = *p* < 0.05; between sesamin treatment and IL-1β pre-treatment alone: * = *p* < 0.05

After the suppression of aggrecan core protein (*ACAN*) gene expression (Fig. 1a) and decrease of GAGs release from HAC pellets (Fig. 1d) had been demonstrated, the ameliorative effect of sesamin on the chondroitin sulfate synthesis was explored. The expression of CSPGs synthetic genes was investigated in the presence of various concentrations of sesamin (0-1 μM) after a 3 day pre-treatment of 10 ng/ml IL-1β. The expression of CSPGs synthesis genes (*ACAN, XT-1, XT-2, CHSY1* and *ChPF*) were analyzed on days 1, 7, 14 and 21 by real-time RT-qPCR. The results on day 1 through day 14 clearly showed that the expression of all CSPGs synthesis genes was suppressed in the HAC pellets pre-treated with 10 ng/ml IL-1β. Sesamin reversed the effects of IL-1β by upregulation of all CSPG synthesis genes *(ACAN, XT-1, XT-2, CHSY-1* and *ChPF)* compared to pellets pre-treated with IL-1β alone (Fig. 3). In addition, the expression patterns of CSPG synthesis genes in the two groups were different. The highest expression of CHSY*-1* and *ChPF* were observed on day 7, while the highest expression of *ACAN, XT-1* and *XT-2* were observed on day 14. Considering the highest expressions of each gene, the results show that sesamin up regulated expression of all of CSPG synthesis genes expression in a dose dependent manner when compared to the IL-1β pre-treatment alone and the significantly upregulation was observed at 1 μM of sesamin (Fig. 4).

### Effect of sesamin on the level of GAGs secretion in IL-1β-induced HAC pellets

The effect of sesamin on GAGs production in IL-1β-induced chondrocytes was determined by pre-treatment of HAC pellets with 10 ng/ml of IL-1β for 3 days followed by various concentrations of sesamin for 21 days. The culture media were collected every 2 to 3 days to measure GAGs release by DMMB assay. Secretary GAGs in culture media of IL-1β treated HAC pellets decreased compared to control pellets. With sesamin post-treatment, production and synthesis of GAGs in IL-1β pre-treated pellets gradually recovered in a dose and time dependent manner (Fig. 4a). In a comparison between the IL-1β pre-treatment alone group and the 21 day sesamin post-treatment group, there was strong evidence that in the presence of sesamin GAGs release was dramatically increased in a dose and time dependent manner. These results correlate with CSPGs synthesis gene expression (Fig 3). It was evident that by 21 days post-treatment sesamin had effectively restored IL-1β and reduced CSPGs production by increasing the expression of core proteins and glycosyltransferase responsive genes that influence core-protein translation and GAGs synthesis.

In addition to measurement of GAGs released in the condition medium, the accumulated GAGs in the matrix of HAC pellets were also analyzed. HAC pellets were pre-treated with 10 ng/ml of IL-1β for 3 days followed by various concentrations of sesamin for a further 21 days. At day 21, the papain digested pellets were measured for matrix accumulated GAGs by DMMB assay. The GAGs concentration was normalized by DNA content. A slight decrease in GAGs accumulation was detected with IL-1β pre-treatment alone when compared to control. Conversely, GAGs accumulation was increased by sesamin post-treatment in a dose dependent manner; a significant increase was observed at 0.5-1 μM of sesamin when compared to IL-1β pre-treatment alone (Fig. 4b). GAGs accumulation in pellet matrix supported the CSPGs synthesis gene expression and secretary GAGs in culture media.

The concentration of GAGs both in culture media and in pellet matrix was expressed as the total of the GAGs. The decrease of total GAGs with IL-1β pre-treatment was reversed by sesamin post-treatment in a dose dependent manner (Fig. 4c). This result demonstrates the promotion effect of sesamin on GAGs and CSPG synthesis in IL-1β induced HAC pellets.

### Effect of sesamin on decorin gene expression in IL-1β induced HAC pellets measured using real-time RT-qPCR

**Fig. 5:**
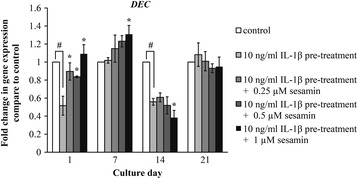
The effects of sesamin on decorin gene expression in IL-1β pre-treated HAC pellets. Data presented are the mean ± S.D. of three independent cultures. Statistical significance of the difference between IL-1β pre-treatment alone and untreated (control): * = *p* < 0.05; between sesamin treatment and IL-1β pre-treatment alone: ^#^ = *p* < 0.05

In addition to aggrecan, another predominant CSPG found in cartilage matrix is decorin. This molecule is much smaller than aggrecan (approximately 40 kDa) and consists of either chondroitin sulfate (CS) or dermatan sulfate (DS). The synthesizing process of decorin in the CS linkage region uses the same set of CSPG synthetic genes with the exception of the decorin core protein (*DEC*) and dermatan sulfate epimerase (*DSE*) genes for core protein synthesis and for DS linkage region synthesis, respectively. Investigation of the effects of IL-1β and sesamin on decorin gene expression found that IL-1β transient suppressed the expression of decorin core protein only on days 1 and 14, unlike the control (Fig. 5). After suppression, cells responded by increasing expression of *DEC* 1.5 fold in a new cycle at days 7 and 21. Sesamin significantly increased the expression of decorin core protein only on days 1 and 7 with 10 μM sesamin treatment, but not in a dose and time dependent manner as with IL-1β treatment alone.

### Histological evaluation of the effect of sesamin on GAGs accumulation in IL-1β-treated pellet matrix

**Fig. 6:**
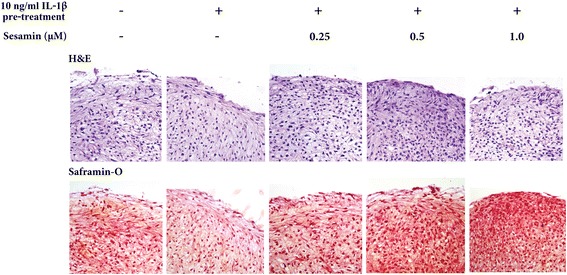
H&E staining (upper panel) and proteoglycan deposition (lower panel) of HAC pellets on day 21. Chondrocyte mophology and matrices were assessed with H&E staining. The sulfated glycosaminoglycan attached to matix proteoglycans was assessed with safranin-O. Representative sections from one donor are shown at 400× magnification

IL-1β pre-treated HAC pellets were collected and histochemical analysis was performed 21 days after treatment with sesamin. HAC pellets were sectioned and stained with either Hematoxylin Eosin (H&E) for analysis of cell morphology or with Safranin-O for analysis of GAGs acumulation (Fig. 6). The distribution of HAC on the surface and in the matrix of the IL-1β pre-treated pellets was normal when compared to the control. The morphology and distribution of chondrocyte in the pellets in the presence of sesamin after pre-treatment of IL-1β were not changed when compared to the control HAC pellets. Safranin-O staining reveals the GAGS acumulation in pellet matrix which can be observed by the intensity of red dye staining. The matrix of HAC plellets of the IL-1β pre-treatment alone group had a pale-red color compared to the control group. In contrast, in the 21 day samples post-treated with sesamin, the intensity of red dye in the matrix of HAC pellets was significantly increased in a dose dependent manner (0.25, 0.5, 1 μM) compared to IL-1β pre-treatment alone group.

## Discussion

We established cartilage inflammatory conditions in a 3D chondrocyte culture system. The primary chondrocyte pellets were created to mimic cartilage structure and inflammation was stimulated with IL-1β. The HAC pellet culture model can be used to investigate the effects of biomolecules, synthetic chemicals or pro-inflammatory cytokines on chondrocytes better than monolayer cultures because chondrocyte phenotypic stability is maintained in this 3D environment [11, 12]. Moreover, long term cultures in this system permit chondrocytes to form and to deposit a well-organized matrix.

We optimized the concentration of IL-1β to stimulate a pathological condition on the chondrocytes by treating the HAC pellets with various concentrations of IL-1β (2.5-10 ng/ml) for 3 days, sufficient to induce a pellet to inflammatory condition. The medium containing IL-1β was then discharged. The IL-1β pre-treated pellets were cultured in IL-1β-free medium for a further 21 days. Culture media was changed and collected every 2-3 days for secretary GAGs analysis and pellets were collected at days 1, 7, 14 and 21 to measure anabolic gene expression (*ACAN)* and catabolic gene expression (*MMP-13* and *ADAMTs4*). One dose of IL-1β pre-treatment effectively induced a pathological condition on the HAC pellets throughout the 21 day culture period. The highest concentration (10 ng/ml) of IL-1β significantly decreased *ACAN* and increased *MMP-13* and *ADAMTs-4* gene expression when compared to the control.

The protein level analysis of IL-1β treated pellet lysates, we found that aggrecan increased in concentration dependent on day1. This reflects both of the intact and degraded forms, which generated by the proteolytic enzymes in HAC pellet. The western blotting analysis showed slightly induction of MMP-13 and ADAMTS-4 when compared to mRNA level as a result of the modulation of translation, secretion and degradation process during the tissue remodeling in the HAC pellet.

Interestingly, IL-1β reduced GAGs production in HAC pellets as indicated by decreased secretary GAGs. Both results indicate the success of IL-1β in promoting the pathological condition in the HAC pellets.

To demonstrate that IL-1β pre-treatment was sufficient to stimulate the inflammation process in the HAC pellet culture system, the protein and mRNA levels of IL-1β pre-treated HAC pellets were analyzed. We found that retention of IL-1β in the cultured medium after pre-treatment was caused by exogenous IL-1β induced endogenous IL-1β synthesis. After changing the media, IL-1β was detectable in the pellet culture medium for entile 21 days the culture period. The level of IL-1β released in the culture medium was significantly higher than the control. This result is in accord with the upregulation of IL-1β gene expression of the IL-1β pre-treated pellets. We have shown that exogenous IL-1β pre-treatment enhances the expression of endogenous IL-1β of HAC pellets both at the gene and the protein level [13].

The expression of IL-1β down-stream target genes (*ACAN* [14], *MMP13* [15] and *ADAMTs-4* ^16^) responded to the level of IL-1β in the condition media. At day 21 in the IL-1β pre-treated HAC pellets, the expression of *ACAN* was not suppressed when compared to days 1, 7 and 14 due to the low levels of IL-1β in the condition media, analogous to the low expression of the *MMP13* gene detected by the presence of low levels of IL-1β in the condition media. The aggrecanase gene (*ADAMTs-4*) when treated with IL-1β increased suddenly at day 14 after declining on day 7 then returning to a slightly higher level on day 21, possibly due to IL-1β autocrine stimulation.

HAC pellets which were pre-stimulated with IL-1β before being cultured in conditional IL-1β free medium still maintained the pathological condition as a result of autocrine stimulation throughout the 21 day culture period. The single dose of exogenous IL-1β may initiate other downstream IL-1β cytokine cascades such as IL-6, IL-8, nitric oxide (NO) and prostaglandin E_2_, exacerbating the inflammation condition and maintaining their effect for the entire 21 days.

There are some limitations of this study. First, we quantified only the amount of IL-1β and thus could not exclude the possible involvement of other cytokines and proteases in the observed IL-1β-mediated matrix reduction. Second, the culture medium consisted of dexamethasone, which is commonly used in cartilage tissue engineering for maintaining the chondrogenic phenotype [17]. The effects of this synthetic glucocorticoid on cartilage matrix turnover are still unclear. It would be interesting to investigate the effects of this compound on the association of IL-1β expression. In addition, in the presence of IL-1β, decreases in the the levels of GAGs secretion in the media were correlated with *ACAN* gene expression. By the Farndale assay, IL-1β decreases GAGs release in a dose and time dependent manner. One of the cartilage-specific biomolecules which contains GAGs is aggrecan. Aggrecan is chondroitin sulfate proteoglycan (CSPG) which is synthesized by chondrocytes [18]. In chondrocyte monolayer cultures and cartilage explants cultures, IL-1β enhances the release of GAGs in culture media when compared to the normal condition [4]. In contrast, in a pellet culture system, the aggrecan core protein expression is suppressed in the presence of IL-1β which reduces the supply of core protein for glycosaminogycans side chain attachment, subsequently affecting CSPGs production. The glycosaminoglycan chains found naturally in matrix are covalently bound to the core protein of proteoglycans, thus the GAGs level reflects proteoglycan production. This finding is consistent with previous reports. IL-1β induces downregulation of aggrecan and xylosyltransferase-1 expression while it upregulates MMP-13 and ADAMTs expression [14–16, 19, 20]. The most likely explanations for this circumstance are, first, that pellet cultures have a higher ratio of cells in extracellular matrix than monolayer and explants cultures, providing sufficient cells to produce matrix-degrading enzymes and to decrease synthesis metabolism [21]. Second, the low media volume relative to the number of cells may result in secreted enzymes being more concentrated in the extracellular matrix [22]. Third, changing the media every 2 to 3 days instead of daily could concentrate the proteolytic enzymes and thus degrade the matrix [20]. A previous model of the anti-inflammatory effect of sesamin on IL-1β-induced porcine cartilage explants have shown that proteoglycan degradation was reduced when sesamin was co-cultured with IL-1β [4]. In that study, sesamin reversed the effects of IL-1β by decreasing degraded GAGs release in explants culture media and the abrogation of uronic acid loss from cartilage tissue. Our study found that sesamin suppresses the autocrine signaling of IL-1β by decreasing IL-1β-induced chondrocyte endogenous IL-1β production. The expression of IL-1β was downregulated both at the mRNA and the protein levels in the presence of sesamin when compared to IL-1β pre-treatment alone.

The highest CSPGs gene expression in normal HAC was on day 14, while in IL-1β induced HAC, the highest expression of *CHSY1* and *ChPF* was on day 7, while for *ACAN, XT-1* and *XT-2* it was on day 14 which differs from a previous study [9]. The difference of CSPGs expression pattern in IL-1β-induced HAC may have been due to chondrocyte compensatory mechanisms which counter IL-1β action [23]. In addition, sesamin might upregulate CSPGs synthesis gene expression by abrogation of endogenous IL-1β and/or by directly upregulating CSPGs synthesis genes. This effect has been found with other phytochemical extracts such as edible bird’s nest [24] and Herbal-Leucine mix [25] that both inhibit IL-1β expression and increase aggrecan synthesis.

To determine whether sesamin specifically effects only aggrecan biosynthesis, the effect of sesamin on another CSPGs gene named decorin was also investigated. Decorin is a smaller matrix biomolecule associated with collagen fibrils in ECM of cartilage. It is a small leucine-rich proteoglycans that consists of a core protein with a dermatan sulfate chain and/or chondroitin sulfate chain attachment. It also modulates cell adhesion to fibronectin and thrombospondin, but not to type I collagen [26]. In this study, no IL-1β transient suppression of the expression of decorin core protein mRNA or alteration of effects of sesamin on decorin core protein were observed. The differences in the response and expression patterns of aggrecan and decorin with IL-1β stimulation may be due to the responsive element on their promoter regions. The aggrecan promoter (choromosome 15q26.1) [27] is spanned by three overlapping SP-1/AP-2 binding sites [28]; in contrast, the decorin promoter region (chromosome 12q23) [29] contains HSF2 and SP-1 sites and has a CRE-like sequence [30].

All the CSPGs biosynthesis results were confirmed by histological analysis. The cytotoxicity and cell morphology of chondrocytes was performed by H&E staining which showed no difference in cell morphology and matrix appearance between IL-1β, IL-1β in combination with sesamin treated pellet sections and the control pellet section. Thus, IL-1β and sesamin treatment had no pathological effect on chondrocyte cells. Analysis of GAGs accumulation in pellet matrix was performed by Safranin-O staining. The results clearly illustrated the lower intensity of Safranin-O staining in the IL-1β pre-treatment alone group compared to the control group. The high Safranin-O intensity was clearly shown in the combination IL-1β and sesamin group compared to the IL-1β pre-treatment alone group in a dose dependent manner. The effect of IL-1β on GAGs accumulation in pellet matrix has been reported in previous studies, where IL-1β exposure to HAC pellets caused an extensive loss of cartilaginous matrix as evidenced histologically by the absence of stained S-GAG and Type II collagen and biochemically by a reduction of GAGs to undetectable levels [23].

In a previous study, sesamin treatment alone increased GAGs accumulation in pellet matrix compared to control conditions and in OA pathological conditions by papain-induced OA rats, showing that sesamin resulted in recovery and increase in the synthesis of cartilage matrix molecules (GAGs and type-II collagen) [4].

This study indicates that sesamin may affect both recovery from inflammatory effects and directly by increasing GAGs synthesis in HAC pellets. Sesamin has previously demonstrated regulation of the chondrocyte catabolic process via suppression of expression of MMPs through the inhibition of IL-1β signaling cascades [4]. It has also shown induction on CSPGs proteoglycan synthesis via enhancement of chondrocyte CSPGs synthetic genes [9].

## Conclusions

This study provides new evidence about the dual effects of sesamin on inflammatory induced chondrocytes through IL-1β expression suppression and through CSPGs synthesis induction, one of the therapeutic targets for OA. Sesamin supplementation can have a synergistic effect on drugs for osteoarthritis treatment that target IL-1β production and processing. However, further studies are needed on the role of sasamin related to signaling pathways of CSPGs synthesis and other targeted biomolecules in extracellular cartilage under both normal and pathological conditions.

## Abbreviations

ADAMTS: a disintegrin and metalloproteinase with thrombospondin motifs
CSPG: chondroitin sulfate proteoglycan
GAG: glycosaminoglycan
HAC: human articular chondrocyte
IL: interleukin
MMP: matrix metalloproteinase
OA: osteoarthritis
